# Synthesis of electrospun polyacrylonitrile- derived carbon fibers and comparison of properties with bulk form

**DOI:** 10.1371/journal.pone.0201345

**Published:** 2018-08-09

**Authors:** Ibrahim M. Alarifi, Waseem S. Khan, Ramazan Asmatulu

**Affiliations:** 1 Department of Mechanical and Industrial Engineering, Majmaah University, Majmaah, Saudi Arabia; 2 Department of Mechanical Engineering, Higher Colleges of Technology, Dubai Men’s College, Dubai, UAE; 3 Department of Mechanical Engineering, Wichita State University, Wichita, United States of America; Institute of Materials Science, GERMANY

## Abstract

This study deals with the fabrication of polyacrylonitrile (PAN) nanofibers via an electrospinning process followed by stabilizing and carbonization in order to remove all non-carboneous matter and ensure a pure carboneous material. The as-spun PAN fibers were stabilized in air at 270°C for one hour and then carbonized at 750, 850, and 950°C in an inert atmosphere (argon) for another one hour. Differential scanning calorimetry and Raman spectroscopy were employed to determine the thermal and chemical properties of PAN. Surface features and morphologies of PAN-derived carbon nanofibers were investigated by means of scanning electron microscopy (SEM). SEM micrograms showed that fiber diameters were reduced after carbonization due to evolution of toxic gases and dehydrogenation. The Raman spectra of carbonized fibers manifested D/G peaks. The Raman spectroscopy peaks of 1100 and 500 cm^-1^ manifested the formation of γ phase and another peak at 900 cm^-1^ manifested the formation of α-phase. The water contact angle measurement of carbonized PAN fibers indicated that the nanofibers were superhydrophobic (θ > 150^o^) due to the formation of bumpy and pitted surface after carbonization. In DSC experiment, the stabilized fibers showed a broad exothermic peak at 308°C due to cyclization process. The mechanical andThermal analysis was used to ascertain mechanical properties of carbonized PAN fibers. PAN-derived carbon nanofibers possess excellent physica and mechanical properties and therefore, they may be suitable for many industrial applications such as energy, biomedical, and aerospace.

## Introduction

Carbon fibers (CFs) are synthesized from three precursors such as rayon, polyacrylonitrile (PAN), and mesophase pitch [1]. Rayon was the original precursor for carbon fibers [2–3]. The low productivity of rayon-based CF has impeded its application as a precursor for the production of commercial CFs. Therefore, PAN and mesophase pitch has been preferred as precursors for carbon fibers production in commercial applications. However, mesophase pitch-based CFs possess inferior mechanical properties especially tensile strength compared to PAN-based CFs. Therefore, PAN has been excessively used materials for carbon fibers precursor for almost three decades [2].In order to synthesize PAN-based carbon nanofibers, three stages are essentials: electrospinning, stabilizing, and carbonization. First, PAN fibers are produced via an electrospinning process then stabilized in air at 270°C, which causes cyclization, dehydrogenation and oxidation of PAN fibers [1]. During stabilization, PAN molecules cyclized to transform into an infusible ladder-like structure, which leads to the conversion of C ≡ N bonds into to C = N bonds and crosslinking between PAN molecules occurs [4–5]. Cyclization is an exothermic process, during which large amount of heat is released that promote cyclization of nitrile group in PAN [5]. Additionally, the nitrile group present in PAN have substantial dipole-dipole interactions, which render high cohesive energy density and stiffness in PAN Chain, thereby resulting in high tensile strength [6]. During stabilization, PAN precursor forms a ladder structure that can withstand higher temperature and increases carbon yield. Stabilization is followed by carbonization, the temperature range of carbonization is from 900°C-1000°C in an inert atmosphere. The ladder structure formed during stabilization further cross-links and forms a turbo-static carbon structure. Non-carbon atoms are released and the final carbon content is around 90% after carbonization.

The electrospinning process is similar to drawing process except for the use of electrical forces rather than mechanical or shearing forces. Electrospinning is a technique that utilizes a strong electrostatic filed to draw polymeric solution into ultrafine fibers in a very short time. When a viscous polymeric solution or melt is charged with a high electrostatic potential, the electrostatic forces developed oppose surface tension. The electrostatic field induced charges in the polymer solution. These charged ions move in response to applied field towards the collector screen having opposite polarity, thereby transferring tensile forces to polymer solution. At the tip of capillary tube, the pendant drop takes the shape of a hemispherical drop, generally referred to as **Taylor Cone** in the presence of an electrostatic filed. When the intensity of electrostatic filed overcomes surface tension of the polymer solution, a jet is emanated from the Taylor Cone, which travels linearly for some distance, called jet length, and then experience whipping motion or twisting motion, which is commonly referred to as bending instability of the electrified jet [7]. The bending instability makes fibers very long and reduces the fiber diameter from micron size to nanosize. Evaporation of solvent occurs during jet flight from capillary tube to collector screen. The fibers are collector from the collector screen and dried in an oven for 1-2h at 60°C. The objectives of this study are to produce PAN-derived carbon fibers via electrospinning followed by stabilizing and carbonizing and investigate their thermal, chemical and surface properties by employing differential scanning calorimetry, Raman spectroscopy and contact angle measurement.

## Experimental

### Materials

Polyacrylonitrile (PAN) (CAS No. 25014-41-10) having a molecular weight of 150 kg/mole and dimethylformamide (DMF) (CAS no. 68-12-2, 99.8%) were purchased from Sigma-Aldrich and used without any further purification. Uncured pre-preg carbon fibers composite peel plies were provided from a local store to fabricate carbon fiber composites in a vacuum oven. Pre-preg carbon fiber composite panels (5320–1) were fabricated using a specimen size of 17.78 x 50.8 x 1.401 mm.

### Method

PAN was dissolved in DMF at a 90:10 weight ratio, and the mixture was subjected to shear mixing at 500 rpm for 1 h. Special care was taken to ensure a homogeneous blend of the mixture (PAN polymeric solution). The mixture was then transformed to a 10 ml syringe with an inside diameter of 0.5 mm. Nanofibers were fabricated using the electrospinning technique with 25 kV at a feed rate of 1 ml/h and a spinneret-to-collector distance of 25 cm. The as-produced nanofibers were later converted to carbon nanofibers by stabilizing them in an oxygen atmosphere at 270°C for 1 h followed by carbonization. The carbonization of PAN fibers was performed at three different temperatures in an inert (argon) atmosphere for 1h. the carbonization tempertaures were 750, 850, and 950°C.The heating ramp rate was 5°C/min [8]. After the first heat treatment, the non-carbon elements, such as hydrogen, oxygen, nitrogen, and sulfur, were eliminated and released as volatile matter, leaving behind a high-carbon-content nanofiber.

**Fig 1:**
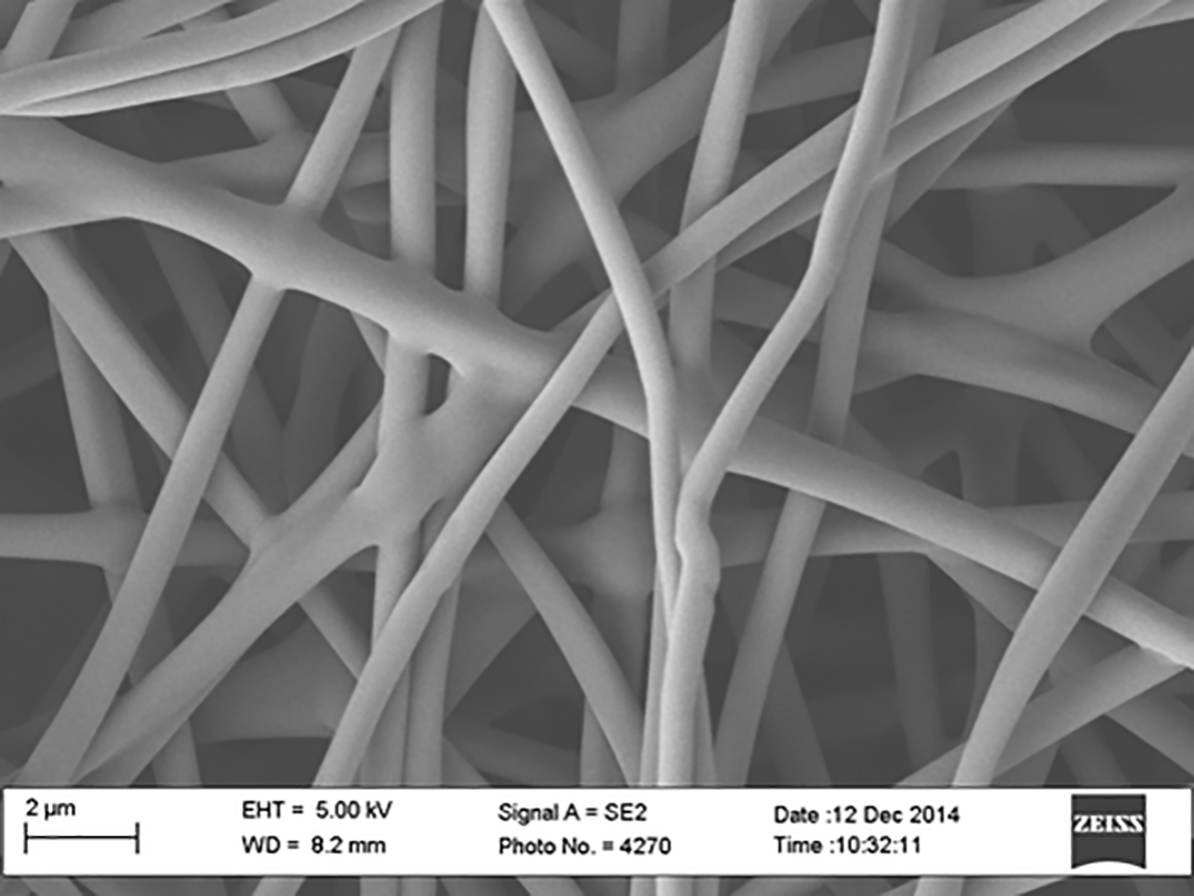
SEM image of PAN nanofibers without carbonization. https://doi.org/10.6084/m9.figshare.6803663.v1.

**Fig 2:**
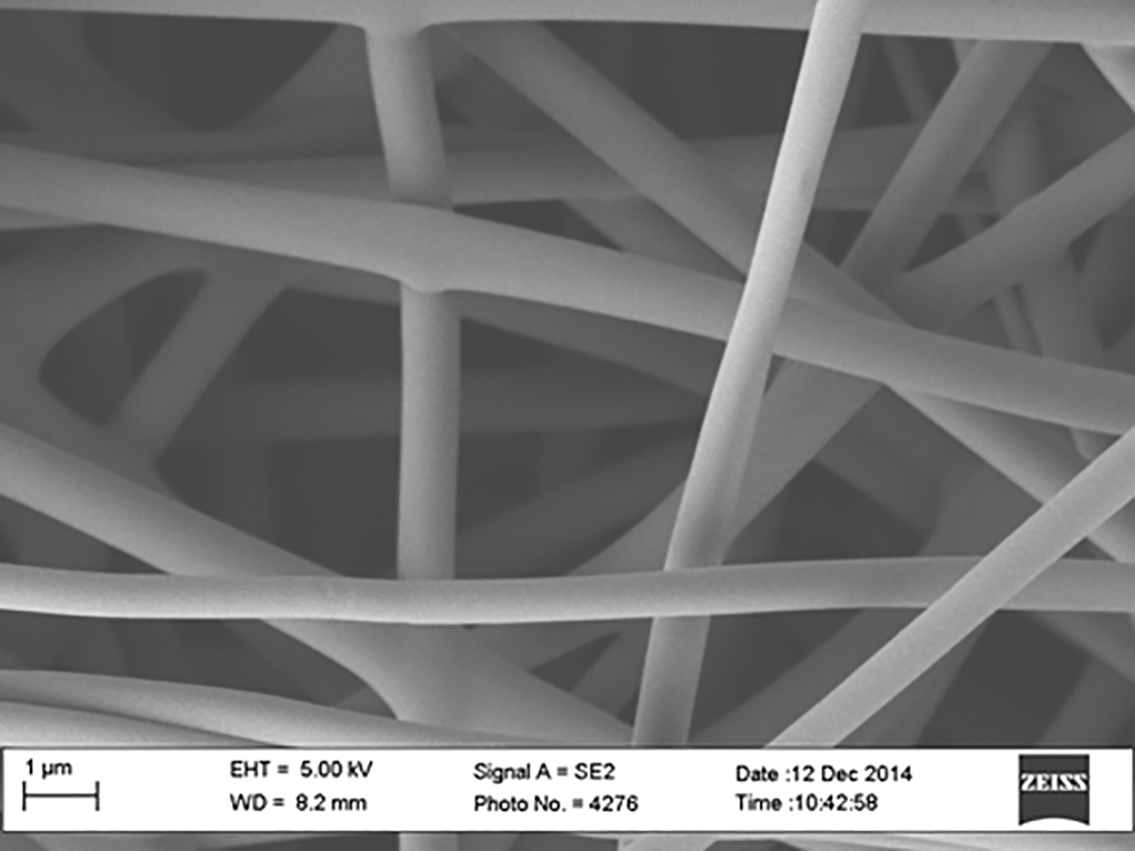
SEM image of PAN nanofibers after carbonization at 750°C. https://doi.org/10.6084/m9.figshare.6803750.v1.

**Fig 3:**
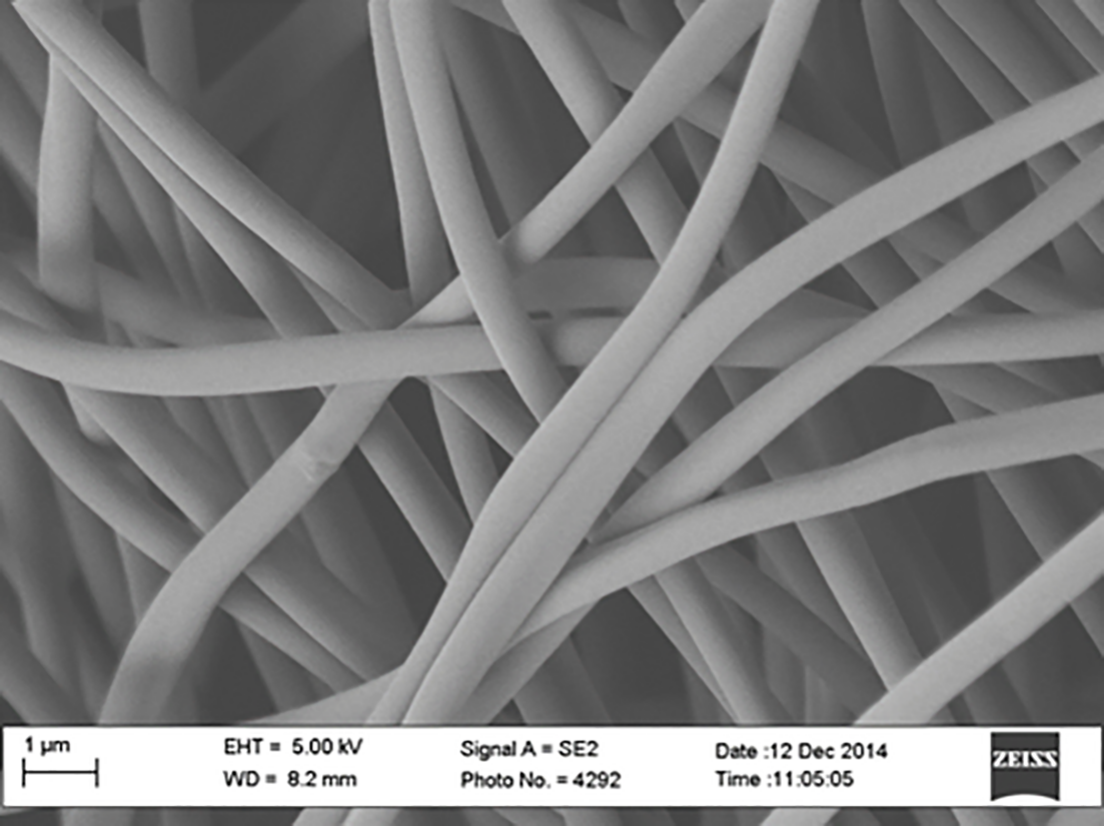
SEM image of PAN nanofibers after carbonization at 850°C. https://doi.org/10.6084/m9.figshare.6803759.v1.

**Fig 4:**
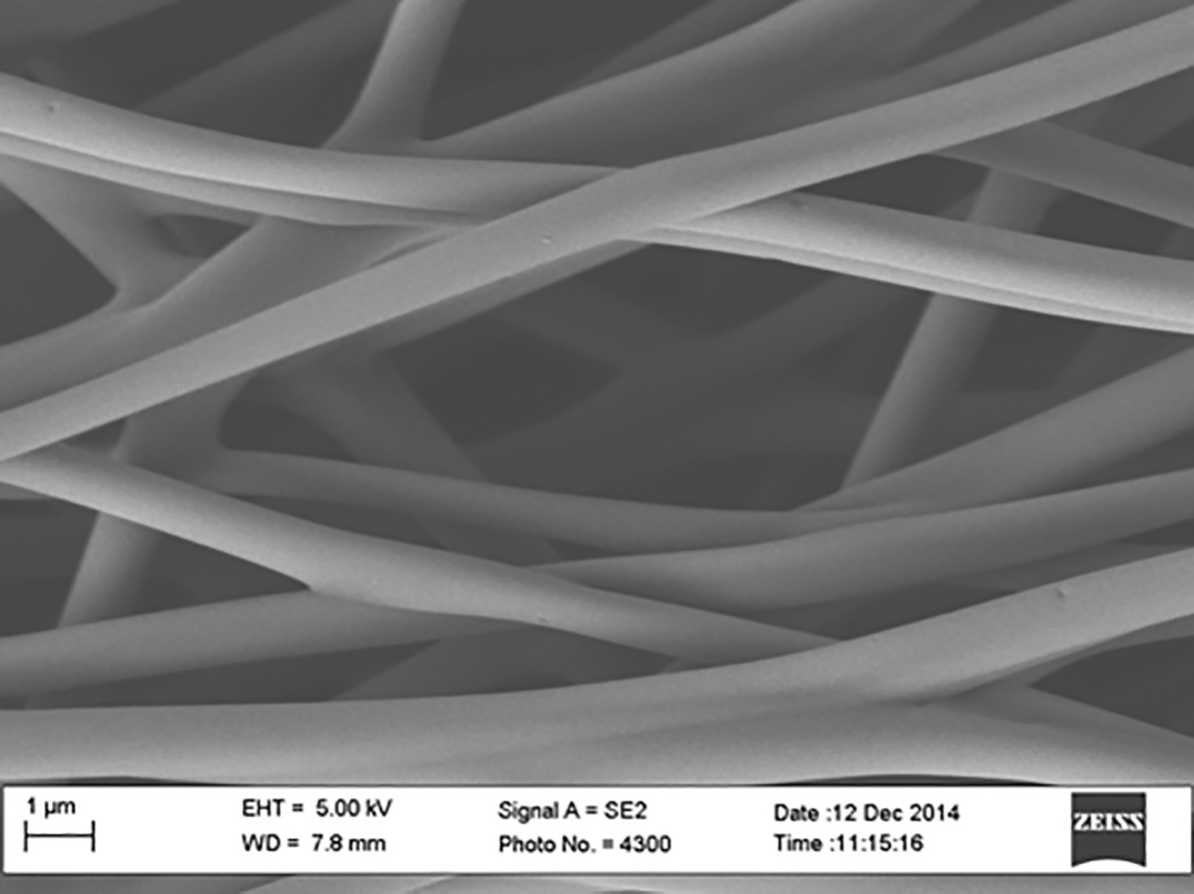
SEM image of PAN nanofibers after carbonization at 950°C. https://doi.org/10.6084/m9.figshare.6803765.

A pre-preg technique was employed to fabricate carbon fiber composite panel (5320–1) having 17.78 x 50.8 x 1.401 mm dimension and incorporated with carbonized PAN nanofibers as the top layer. Pre-preg carbon fibers of ten peel plies were laid up at 0, 45-, -45-, and 90 degree stacking sequences on a flat and smooth aluminum (Al) mold, and then a carbonized electrospun PAN nanofiber mat was placed on top of the last ply prior to vacuum curing in a vacuum oven. A release agent was sprayed several times on the mold before placing the plies on the mold. Then a release film, cold sheet berating film, and vacuum bag film were applied before sealing with a tacky tape. The PAN-derived carbon nanofiber appeared black in color due to the stabilizing and carbonizing processes. The nanofiber film thickness was about 1.401±0.021 mm. Fig 1 shows the SEM image of PAN fibers before carbonization. Figs 2, 3 and 4 show the SEM images of PAN-derived carbon fibers after carbonization at 750°C, 850°C and 950°C, respectively.

As can be seen in Figs 2, 3 and 4, the fibers maintained their shape and morphology after carbonization. However, the fiber diameters were reduced after carbonization. The average diameter of PAN nanofibers before carbonization was roughly 500±10 nm; however, after carbonization, the diameter was reduced to 480±10 nm for 750°C carbonization, 450±10 nm for 850°C carbonization, and 430±10 nm for 950°C carbonization, respectively. The evolution of volatile compounds and shrinkage caused this reduction in diameter. As can be seen in Figs 2, 3 and 4 that the surfaces of fibers became bumpy or uneven due to evolution of toxic compounds and dehydrogenation. The fiber diameters shrunk after heat treatment. The shrinkage can be divided into the entropic part and chemical part. The entropic shrinkage is caused mainly by the retraction of stretched polymer chains, while the chemical shrinkage is caused by the formations of dense structures after the chemical reactions. Entropic shrinkage constitutes physical changes and is independent of the heating rate. However, chemical shrinkage can be increased with increasing the heating rate.

## Results and discussion

### Thermal behavior of stabilized bulk PAN fibers

**Fig 5:**
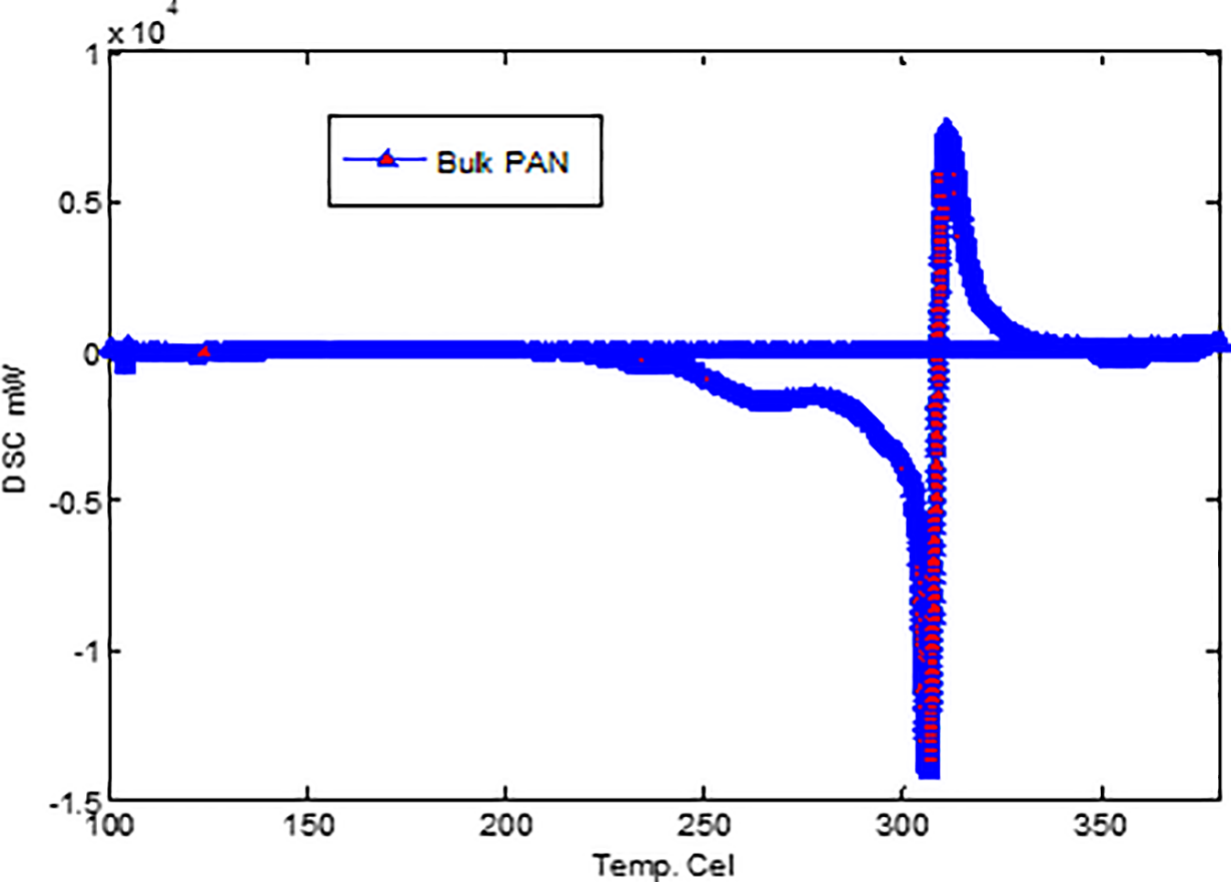
DSC thermogram of bulk PAN polymer. https://doi.org/10.6084/m9.figshare.6803777.v2.

**Fig 6:**
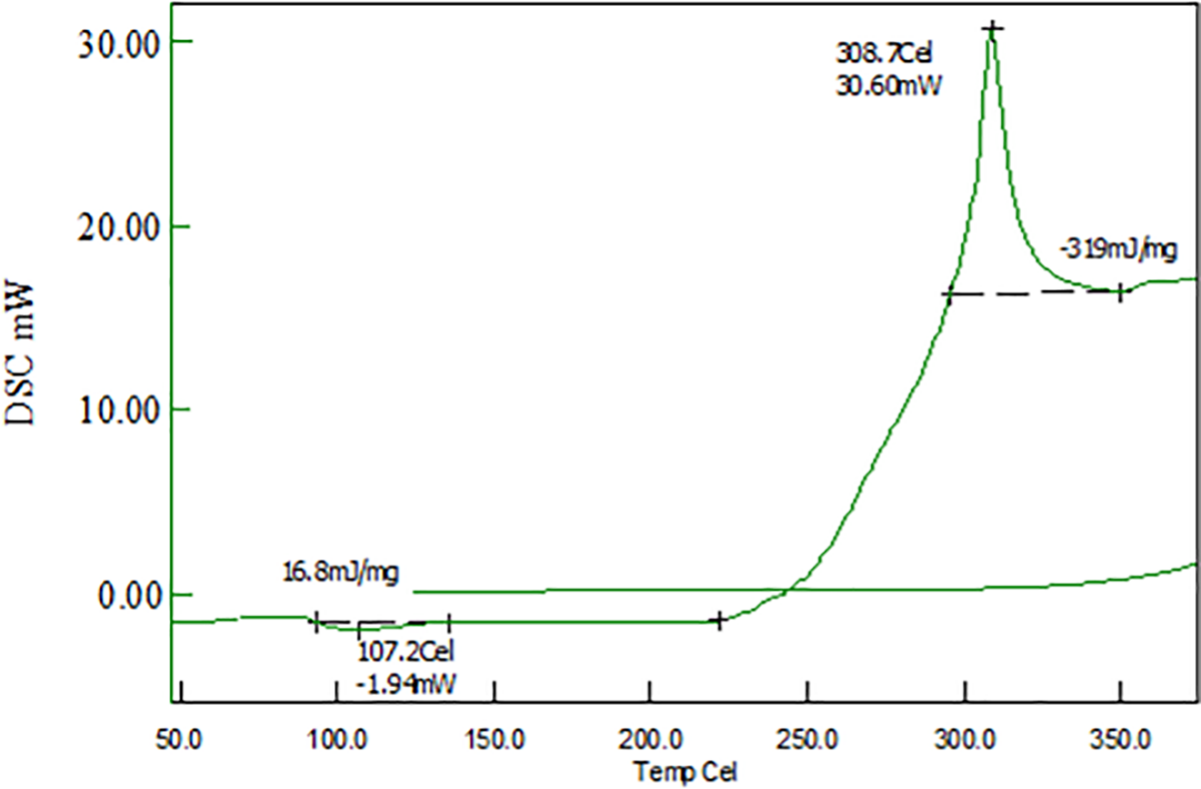
DSC thermogram of stabilized PAN fibers. https://doi.org/10.6084/m9.figshare.6803786.v1.

A Q1000 differential scanning calorimeter (TA Instruments) interfaced to a personal computer (PC) was used to measure the thermal properties of the samples at a heating rate of 10°C/min and a nitrogen (N_2_) flow rate of 50 ml/min. Differential scanning calorimetry is a technique we use to study what happens to polymers when they are heated. We use it to study what we call the thermal transitions of a polymer [9].The samples were sealed in a Tzero™ pan (TA Instruments). A predetermined weight of each sample was used in this experiment. The DSC heat flow process and temperature were calibrated with an indium standard. The stabilization of PAN fibers and bulk PAN polymer was investigated using non-isothermal behavior on a DSC analysis line. A heating rate of 10°C/min in a nitrogen atmosphere was used in this experiment. The bulk PAN sample displayed a sharp peak at 305°C (Fig 5) whereas, the stabilized sample displayed a broadening peak at 308°C (Fig 6).

Broadening of the exothermic peak could be due to the cyclization process. The cyclization of nitrile groups is highly exothermic and leads to fragmentation of the chains owing to heat that builds up rapidly in the sample and does not dissipate rapidly. The DMF molecules obstruct with polymer chain during electrospinning, and as a result, the DSC thermogram of carbonized PAN sample is different than that of bulk PAN sample. Generally, PAN polymer begins to degrade before its melting temperature is reached [10]. The peaks at 305°C for bulk sample and at 308°C for the stabilized sample is due to the presence of nitrile group in PAN [5, 11–12]. Mathur and Jung et al. [13–14] have reported that exothermic peak shift to a lower temperature in electrospun PAN fibers and lower cyclization temperature could be due to enhancement in molecular chain twisting. The chemical processes that take place during heat treatment are cyclization, dehydrogenation, aromatization, oxidation and crosslinking, which results in ladder-like structure [15–17]. In this study, stabilization was performed at 270°C, during which C ≡ C bonds are converted to C = C bonds and crosslinking between PAN molecules occurs, which make PAN infusible. The thermal stability of stabilized PAN is attributed to the ladder-like structure due to cyclization of nitrile group [17]. It is well-known that the heat-capacity difference between PAN fibers and bulk PAN resulted in the heat flow difference in the DSC thermograms. The PAN fibers were cyclized only by a free radical mechanism, revealing one peak [18]. Additionally, the stabilized PAN fibers revealed a higher cyclization temperature than the bulk PAN polymer, suggesting that more thermal energy is needed for cyclization. The chemical processes involved during carbonization are cyclization, dehydrogenation, aromatization, oxidation and crosslinking that generally causes the formation of ladder like structure [16]. During stabilization, PAN molecules absorb oxygen from air and experience chemical changes thereby resulting in the formation of ladder-like structure that no longer melted and therefore retain fiber morphology in the carbonization process [15]. During Carbonization, variety of gases (H_2_O, N_2_ and HCN) are evolved and carbon content increases and fiber diameter decrease [15].

### Raman spectra of carbonized PAN fibers and bulk PAN polymer

**Fig 7:**
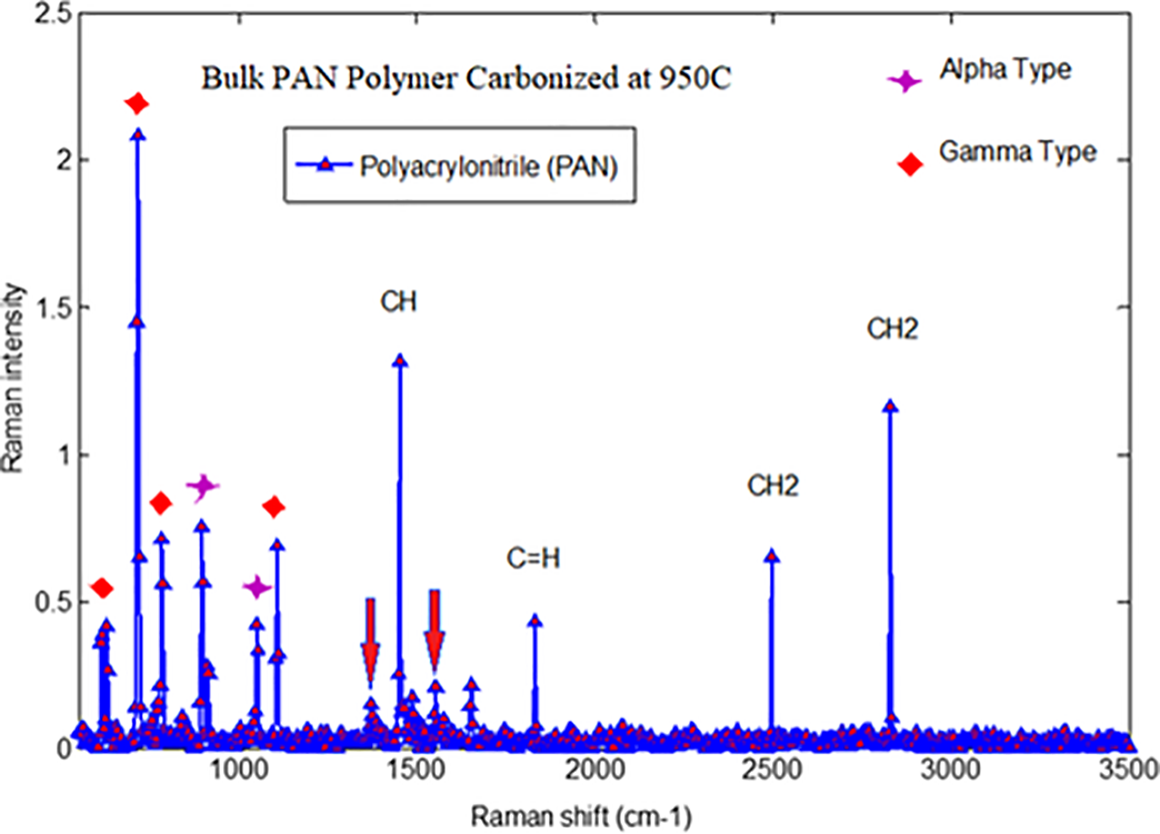
Raman spectra of Bulk PAN Polymer carbonized at 950°C. https://doi.org/10.6084/m9.figshare.6803795.v1.

Fig 7 displays the representative Raman spectra of carbonized bulk PAN investigated in our experiments. The intensity of the peak at 1800 cm^-1^ can be attributed to stabilizing at 280°C. This comes from the tangential vibration of a graphite structure (G-band), and shows additional cross-linking and the formation of a two-dimensional graphitic structure [19]. The Raman spectroscopy peak around 1100 and 500 cm^-1^ indicates the formation of the γ-phase of the carbonized PAN fibers. A Raman spectroscopy peak at 900 cm^-1^ indicates formation of the α-phase.

The highest stretching peak was observed at 2,500, and 2,800 cm^-1^ peaks indicated the existence of CH_2_-group. C = H vibration peaks repeated then appeared at 1,800 cm^-1^. There are two broad overlapping peaks centered approximately at 1340 cm^-1^ and 1580 cm^-1^. They are well-known D and G peaks, respectively. The Raman spectra of carbonaceous materials have two characteristic bands, one centered at 1340 cm^-1^ and appropriately termed as “D-band” and it is related to disordered turbostratic structures and the other centered at the wavenumber of 1580 cm^-1^and termed as “G-band” and it is related to ordered graphite structures [19]. Marx et al. [20] reported that 3D interconnected carbon foam (Aerographite) exhibits two peak, one at 1330 cm^-1^(D-band) and another at 1580 cm^-1^(G-band). Both bands demonstrates different phenomena in carbon structures. D-band describe lattice defects, stacking faults and hybridization, whereas, the G-band describes sp2 hybridization of carbon [20]. As can be seen in Fig 7, showed a peak at 1,500 cm^-1^ (D-peak) characterized the disorderly materials structure of PAN [16]. The G and D peaks increase as the carbonization temperature increases, indicating that the higher carbonization temperature facilitates the arrangement of carbon from a disorderly to an orderly state [21]. The higher carbonization temperature facilitates the formation of a graphite phase [22]. The “G- band” is referred to graphite phase whereas, “D- band” is related to turbulence structure. The ratio of these bands is commonly known as “R-value,” which indicates graphite crystallites in carboneous materials [23].The ratio of the integrated amounts of the “D peak” and “G peak,” designated by $R=\frac{L_{D}}{L_{G}}$, decreases with temperature [23]. Knight and White [24] demonstrated that R behavior depends on the *in* plane behavior graphitic crystallite size La:

$$\mathrm{Crystallite}=\frac{4.4}{R}$$

**Fig 8:**
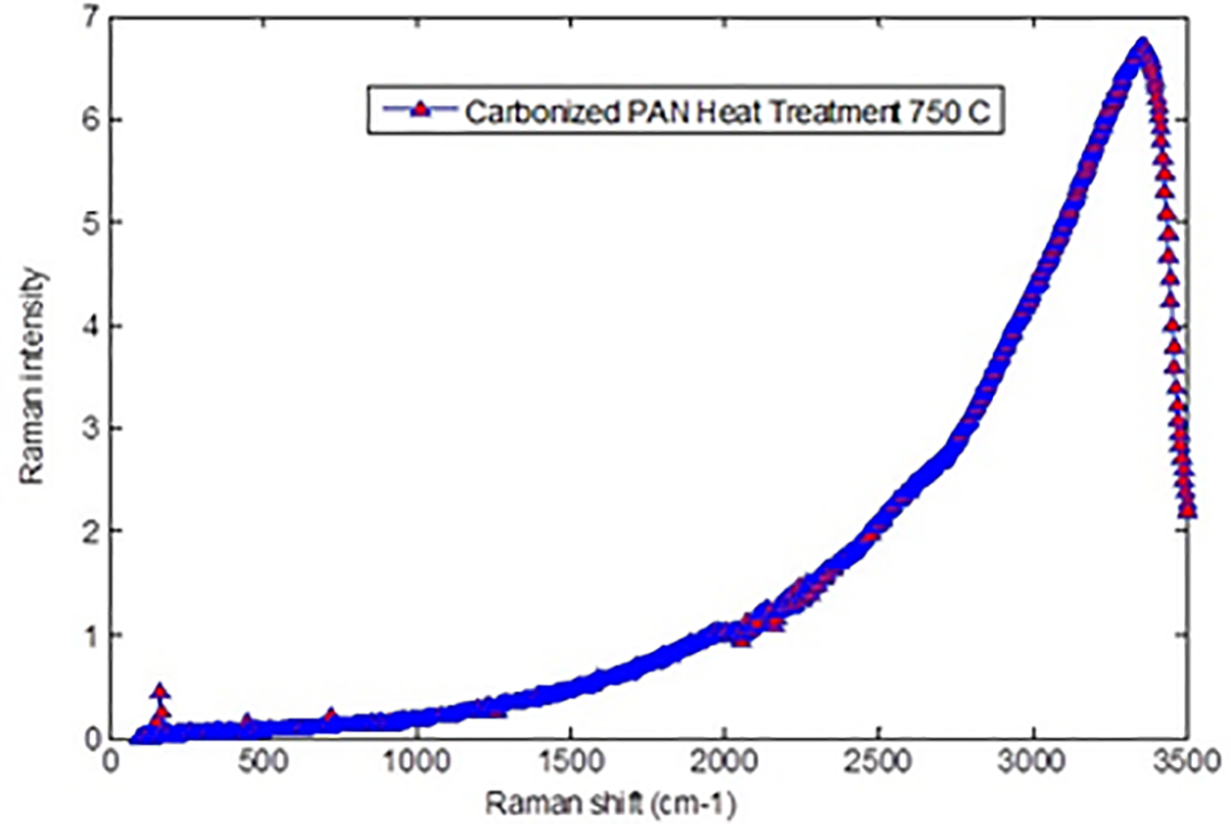
Raman spectra verified of Polyacrylonitrile PAN fibers carbonized at 750°C. https://doi.org/10.6084/m9.figshare.6803819.v1.

**Fig 9:**
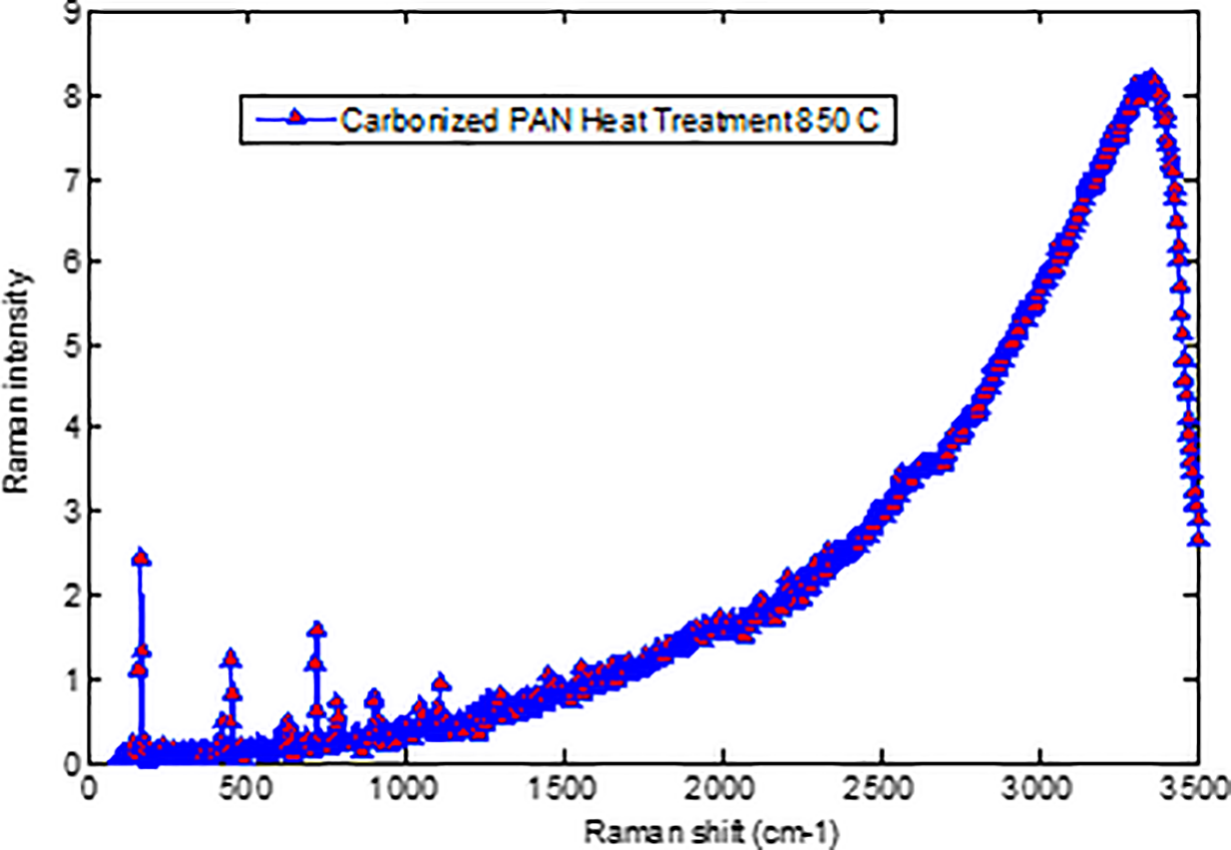
Raman spectra verified of Polyacrylonitrile PAN fibers carbonized at 850°C. https://doi.org/10.6084/m9.figshare.6803834.v1.

**Fig 10:**
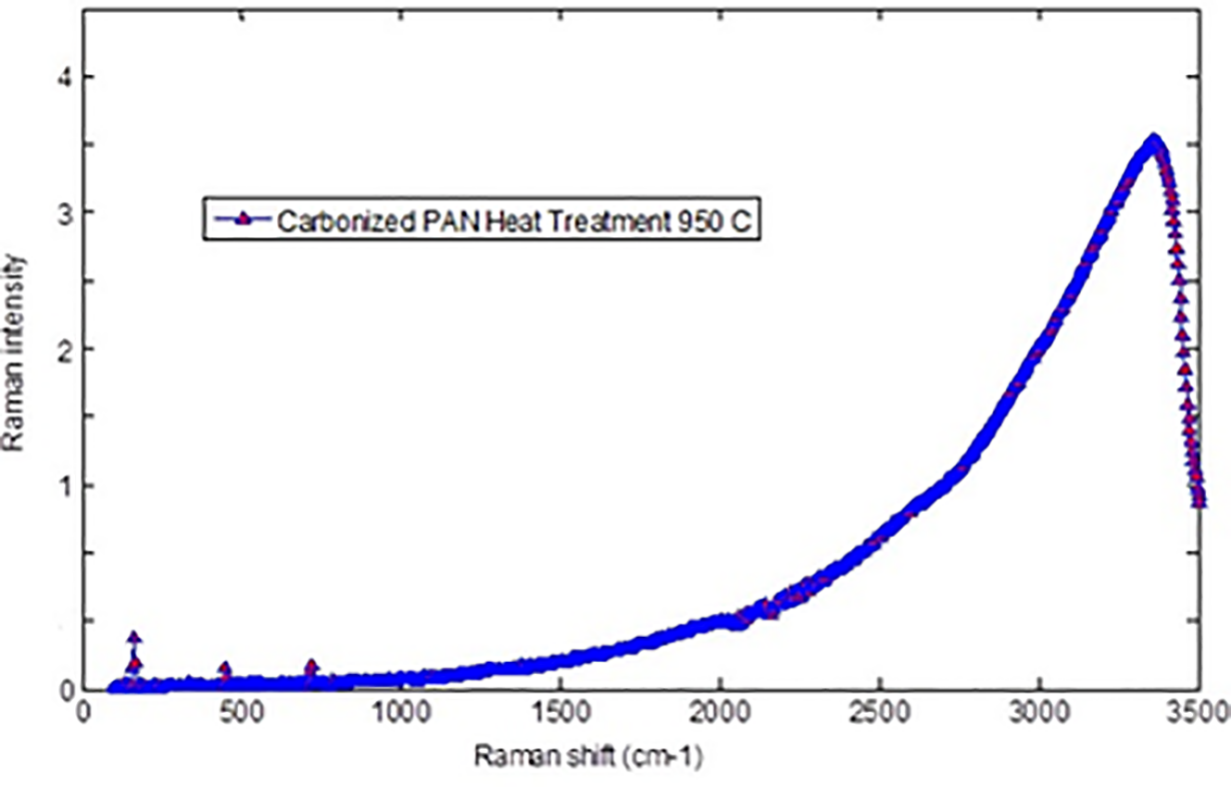
Raman spectra verified of Polyacrylonitrile PAN fibers carbonized at 950°C. https://doi.org/10.6084/m9.figshare.6803840.v1.

By using this mathematic equation, the in-plane graphitic crystallite size corresponding to different temperature can be determined. The integrated intensities *L_D_* and, *L_G_*, are proportional to the scattering disorder and ordered sp2 bonding in the irradiated area/their mole fraction, respectively [25]. The decrease in R-value due to the rise in temperature is an indication of excessive graphite mole fraction [26]. Figs 8, 9 and 10 show Raman spectra of PAN-derived carbon fibers carbonized at 750°C, 850°C, and 950°C, respectively. As shown in Fig 9, the Ramn spectra of carbonized PAN fibers at 850°C exhibit that the “G band” was due to C = C was stretching vibrations behavoir in graphite phase and “D- band” was due to the turbulence and disordered carbonaceous materials.

As can be seen in Figs 8, 9 and 10 (G/D) peaks were increased as the carbonization temperature increased. All three figs show a carbonization line with no peak since all samples are rich in carbon content. All three figures show a carbonization line with no peak since all samples are pure carbon or black samples. Therefore, the black samples show the steady line behavior with no peaks for carbonized PAN nanofiber composite samples. There is an apparent sharp peak, corresponding to (002) plans for carbonized PAN [16].

### Surface characterization of carbonized PAN fibers

**Fig 11:**
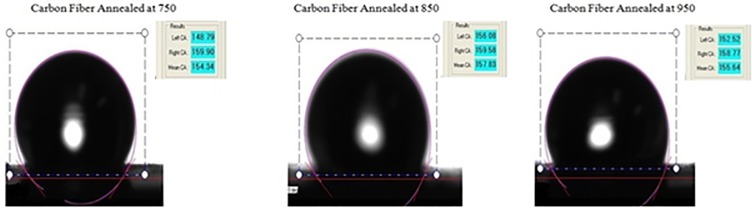
Static water contact angle values of PAN-derived carbon fibers at different carbonization temperature. https://doi.org/10.6084/m9.figshare.6803849.v1.

**Fig 12:**
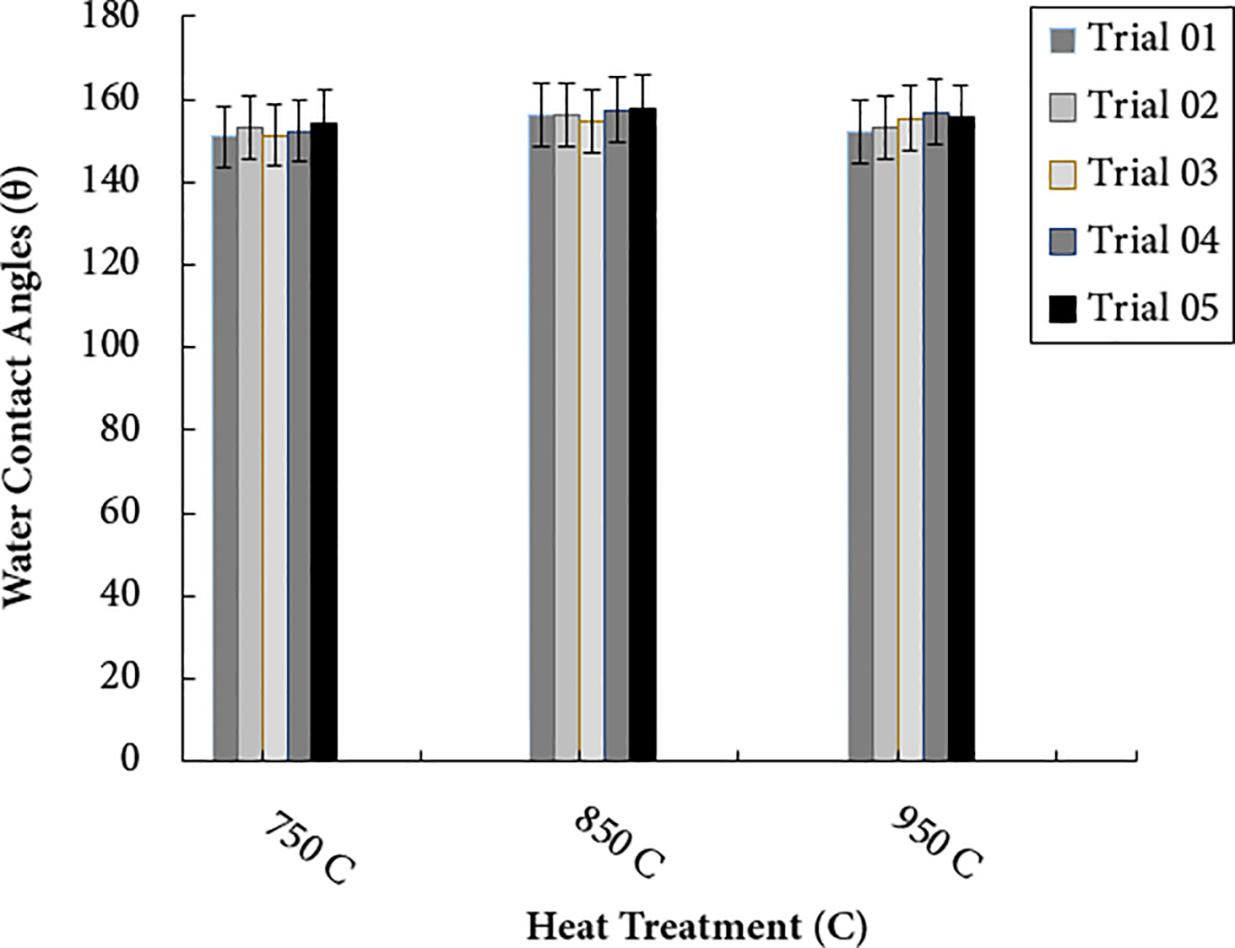
Histogram of static water contact angle values after at 750°C, Carbonization at 850°C and Carbonization at 950°C. https://doi.org/10.6084/m9.figshare.6803858.v1.

In this study, static water contact angle values of the carbonized PAN-derived carbon nanofiber samples at 750°C, 850°C, and 950°C were measured employing an optical noticed contact angle goniometer with a CAM100 camera (KSV Instruments Ltd). Fig 11 shows static water contact angles values of PAN-derived carbon surface with different heat treatment temperatures. Fig 12 shows the histogram of static water contact angle values at different carbonization temperature.

Hydrophobic and Hydrophilic are generally used descriptors of solid surfaces [27]. A surface is said to be hydrophobic if it tends not to adsorb water or be wetted by water through making a static contact angle greater than 90 degrees. A surface is said to be hydrophilic if it tends to adsorb water or be wetted by water through making a static contact angle less than 90 degrees [27]. Superhydrophobic surfaces manifest high contact angle (>150^o^) and low contact hysteresis [28]. Solid surfaces with a very low water contact angle (~ 0) are commonly referred to as superhydrophilic. The terms describe the interaction of the boundary layer of a solid phase with liquid or vapor water. A surface is said to be wetted if water spreads over the surface evenly without the formation of droplets, such a surface is termed as hydrophilic [27]. However, water forms distinct droplets on hydrophobic surface, which roll off easily without wetting the surface. In this study, the water contact angles of carbonized PAN samples were measured (Fig 11). The main goal of measuring water contact angle of PAN carbonized samples was to determine whether the carbonization at different temperatures made PAN samples hydrophobic or hydrophilic. As is seen in histogram (Fig 12) that all the PAN carbonized samples are superhydrophobic. The average water contact angle for PAN samples carbonized at 750°C was 155^o^, for PAN samples carbonized at 850°C was 159^o^ and for PAN samples carbonized at 950°C was 160^o^, respectively. It is well known that PAN is a hydrophobic polymer, and after carbonization the contact angle varies due to thermal expansion, evolution of volatile compounds and dehydrogenation. Water droplets on hydrophilic surfaces are either absorbed or spread evenly and exhibit a very low contact angle. Whereas, water droplets on hydrophobic surfaces are stick or stay on the surface exhibiting a very high contact angle. Both superhydrophobicity and superhydrophilicity of solid surfaces are based on surface chemistry (surface energy) and surface roughness. The wettability of solid surfaces is determined by the surface energy and surface smoothness. The surface microstructure and surface chemistry determines whether the droplet of water will roll off or spread evenly on the surface. As can be seen in Fig 1, the surface morphology completely changed after carbonization. The surface appeared as bumpy or pitted after carbonization due to evolution of volatile compound and water vapors during heat treatment thereby making the surface rougher. The presence of DMF in PAN fibers, which evaporated during heat treatment leaving behind a rough and porous microstructure. Wettability depends on the surface free energy and surface roughness and these factors increased during carbonization thereby resulting in an increased in water contact angle.

PAN fibers are semi-crystalline, with some degree of polarity due to radicals in the chain structure, which may affect the water contact angle values. Prior to the carbonization, the PAN nanofibers provided a water contact angle of about 95°. PAN has the tendency to absorb water under normal conditions, and also absorb some moisture from the atmosphere, as well. However, during the carbonization process, the surface chemistry and surface morphology of the PAN fibers are significantly altered. Specifically, the pores and beads in the material are diminished, and the polarity due to the radicals attached to the main chain is eliminated as non-carbonaceous compounds are released.

### Mechanical properties test of CFs pre-preg composite

**Fig 13:**
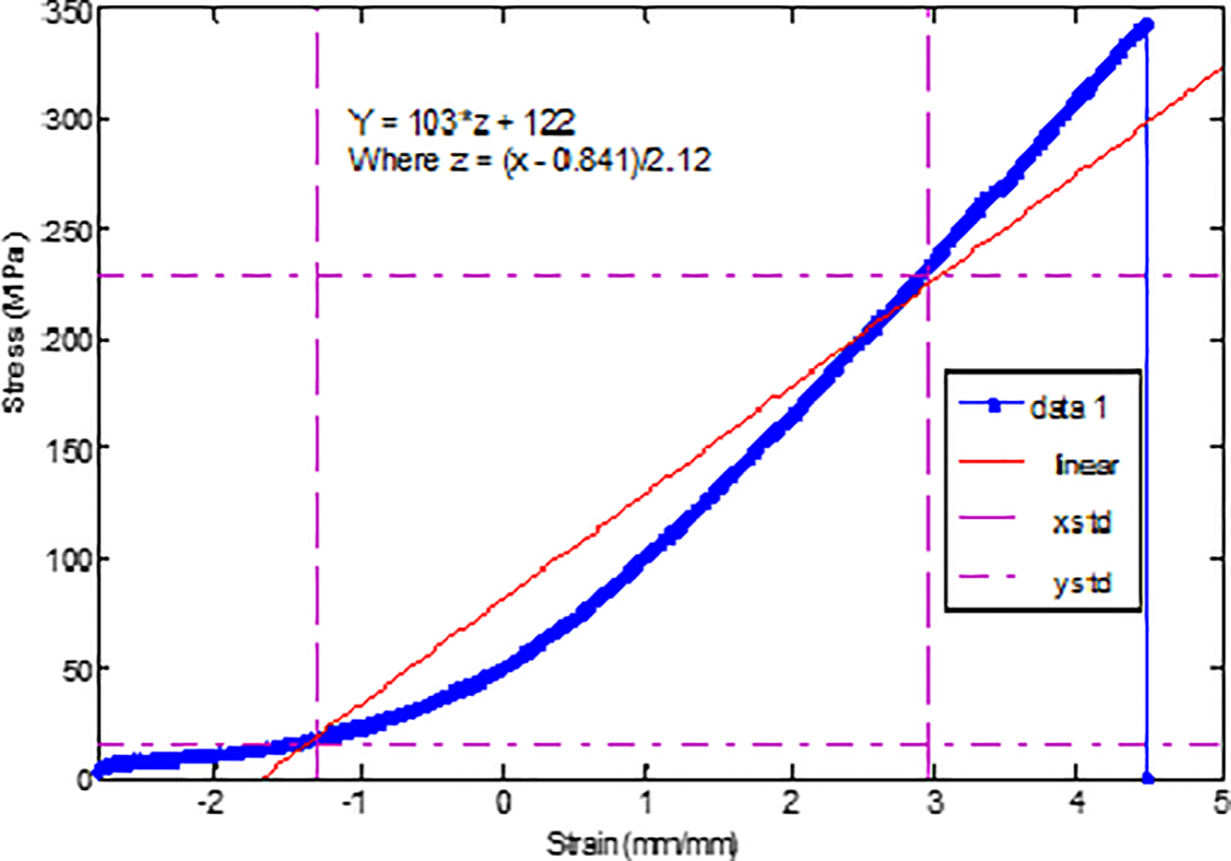
Stress vs. strain behavior of carbon composite with a nano mat of PAN-derived carbon fiber at the top of assembly. https://doi.org/10.6084/m9.figshare.6803873.v1.

Fig 13 shows the stress-strain curve of carbon fiber composite panel with PAN-derived carbon nanofiber nano mat is placed on the top of composite assembly. As is seen in Fig 13, the carbon composite exhibited maximum stress of 350 MPa. Failure analysis was done to validate the result. The carbon composite assembly deformed almost linearly until fracture occurred at 350 MPa. The strain was recorded at 4.5 mm/mm at failure. The carbon composites generally exhibit linear behavior. However, placing a nano mat of PAN-derived carbon fiber slightly changed linear behavior [29]. Table 1 shows mechanical properties of carbon composite assembly with PAN-derived carbon nano mat at the top of assembly at 750°C, 850°C, and 950°C carbonization temperature.

**Table 1:** Mechanical properties of carbon composite assembly with PAN-derived carbon nano mat at the top of assembly at 750°C, 850°C, and 950°C carbonization temperature.

Mechanical Properties of Composite with Carbonized CFs Electrospun PAN Nanofibers				
CFs Composite Specimens	Elastic Modulus (MPa)	Peak Load (N)	Ultimate Stress (MPa)	Strain (mm/mm)
750°C	3913.786	24401.950	342.9	0.032
850°C	4546.988	23882.567	350.6	0.085
950°C	4326.986	23871.826	332.8	0.047

https://doi.org/10.6084/m9.figshare.6803861.v2

Table 1 provides simulation analysis of carbon fiber composite panel with PAN-derived nano mat at the top of the sequence.

## Conclusions

Electrospun nanofibers were produced via an electrospinning process and used as a precursor to produce PAN-derived carbon nanofibers. The electrospun PAN fibers were subjected to stabilization followed by carbonization processes to produce PAN-based carbon nanofibers. The Raman spectroscopy peak around 1100 and 500 cm^-1^ indicated formation of the γ-phase of the carbonized PAN fibers. Likewise, a Raman spectroscopy peak at 900 cm^-1^ indicated formation of the α- phase. The G and D peaks increased as the carbonization temperature increased, indicating that the higher carbonization temperature facilitates the arrangement of carbon from an amorphous to crystalline state. DSC studies showed that the PAN fibers are cyclized only by a free radical mechanism, revealing one peak. Moreover, the stabilized PAN fibers revealed a higher cyclization temperature than the bulk PAN polymer, thus suggesting that more thermal energy is needed for cyclization. These studies also showed that the PAN-derived carbon fibers are rich in carbon content and have excellent thermal stability, so these fibers can be useful for structural health monitoring (SHM), as well as lightning strike and electromagnetic interference shielding applications. Shear stress tests were applied to composite panels to characterize the mechanical properties of novel nanomaterials. The water contact angle measurements revealed that the all the samples were superhydrophobic.These nanomaterials and methods could widely open up many possibilities to developing highly sensitive SHM devices and sensors for composite aircraft and wind turbines, as well as other infrastructures.

## Supporting information

S1 File Supporting information file corresponding to Fig 5. (XLSX)

S2 File Supporting information file corresponding to Fig 7. (XLSX)

S3 File Supporting information file corresponding to Fig 10. (XLSX)

S4 File Supporting information file corresponding to Fig 13. (XLSX)
